# Association between homologous recombination repair gene mutations and response to oxaliplatin in pancreatic cancer

**DOI:** 10.18632/oncotarget.24865

**Published:** 2018-04-13

**Authors:** Tomohiro Kondo, Masashi Kanai, Tadayuki Kou, Tomohiro Sakuma, Hiroaki Mochizuki, Mayumi Kamada, Masahiko Nakatsui, Norimitsu Uza, Yuzo Kodama, Toshihiko Masui, Kyoichi Takaori, Shigemi Matsumoto, Hidehiko Miyake, Yasushi Okuno, Manabu Muto

**Affiliations:** ^1^ Department of Medical Oncology, Graduate School of Medicine, Kyoto University, Kyoto, Japan; ^2^ Biomedical Department, Mitsui Knowledge Industry Co., Ltd., Tokyo, Japan; ^3^ Department of Biomedical Data Intelligence, Graduate School of Medicine, Kyoto University, Kyoto, Japan; ^4^ Department of Gastroenterology and Hepatology, Graduate School of Medicine, Kyoto University, Kyoto, Japan; ^5^ Division of Hepato-Biliary-Pancreatic Surgery and Transplantation, Department of Surgery, Graduate School of Medicine, Kyoto University, Kyoto, Japan; ^6^ Clinical Genetics Unit, Kyoto University Hospital, Kyoto, Japan

**Keywords:** BRCA, homologous recombination repair, oxaliplatin, pancreatic cancer, precision medicine

## Abstract

**Objectives:**

We aimed to examine the association between homologous recombination repair (HRR)-related gene mutations and efficacy of oxaliplatin-based chemotherapy in patients with pancreatic ductal adenocarcinoma (PDAC).

**Results:**

Non-synonymous mutations in HRR-related genes were found in 13 patients and only one patient had a family history of pancreatic cancer. Eight patients with HRR-related gene mutations (group A) and nine without HRR-related gene mutations (group B) received oxaliplatin-based chemotherapy. Median progression-free survival after initiation of oxaliplatin-based chemotherapy was significantly longer in group A than in group B (20.8 months vs 1.7 months, *p* = 0.049). Interestingly, two patients with inactivating HRR-related gene mutations who received FOLFIRINOX as first-line treatment showed exceptional responses with respect to progression-free survival for > 24 months.

**Materials and Methods:**

Complete coding exons of 12 HRR-related genes (*ATM, ATR, BAP1, BRCA1, BRCA2, BLM, CHEK1, CHEK2, FANCA, MRE11A, PALB2,* and *RAD51*) were sequenced using a Clinical Laboratory Improvement Amendment-certified multiplex next-generation sequencing assay. Thirty consecutive PDAC patients who underwent this assay between April 2015 and July 2017 were included.

**Conclusions:**

Our results suggest that inactivating HRR-related gene mutations are predictive of response to oxaliplatin-based chemotherapy in patients with PDAC.

## INTRODUCTION

*BRCA1* and *BRCA2* play pivotal roles in DNA homologous recombination repair (HRR), and germline *BRCA1/2* mutations reportedly increase risk of pancreatic ductal adenocarcinoma (PDAC). A large clinic-based cohort study enrolling 306 patients with PDAC reported the prevalence of pathogenic *BRCA1/2* germline mutations to be 4.6% [1]. Moreover, several retrospective studies have indicated that patients with PDAC harboring germline *BRCA1/2* mutations are more sensitive to platinum-based chemotherapy than those without the mutations [2–4].

In addition to *BRCA1/2*, other genes such as *ATM*, *ATR*, and *PALB2* are involved in HRR [5]. In ovarian cancer, both germline and somatic mutations in HRR-related genes are predictive of response to platinum-based chemotherapy [6]. Therefore, it is reasonable to speculate that patients with PDAC harboring mutations in HRR-related genes in their tumor tissues are sensitive to platinum-based chemotherapy. Supporting this idea, Waddell et al. reported that four of five patients with PDAC who were deficient in *BRCA1*/*2* or *PALB2* responded to platinum-based chemotherapy [7].

Oxaliplatin, a third-generation diaminocyclohexane platinum compound, is now commonly used in patients with PDAC [8–12]. However, to date, studies in patients with PDAC investigating prevalence of HRR-related gene mutations in tumor tissues and their association with efficacy of oxaliplatin-based chemotherapy are sparse. Therefore, we aimed to evaluate the association between HRR-related gene mutations identified using a Clinical Laboratory Improvement Amendment (CLIA)-certified multiplex next-generation sequencing (NGS) assay (OncoPrime^TM^) [13] and efficacy of oxaliplatin-based chemotherapy in patients with PDAC.

## RESULTS

### Patient characteristics

Patient characteristics are summarized in Table 1, and characteristics of individual patients are shown in Table 2. The median age was 64 (range 39–81) years.

**Table 1:** Patient characteristics

		Number of Patients (%)		
		HRR-related gene mutation		Total
		(+) *n* = 13	(−) *n* = 15	
Sex				
	Male	7	8	15 (53.6)
	Female	6	7	13 (46.4)
Age, years				
	Median	61	64	64
	Range	44–81	39–74	39–81
	Age ≤ 60 years	6	6	12 (42.9)
Disease status				
	Locally advanced	5	2	7 (25.0)
	Metastatic	5	6	11 (39.3)
	Recurrence	3	7	10 (35.7)
Family history of cancer				
	Yes	8	9	17 (60.7)
	No	3	4	7 (25.0)
	Unknown	2	2	4 (14.3)
Family history of pancreatic caner				
	Yes	1	3	4 (14.3)
	No	10	10	20 (71.4)
	Unknown	2	2	4 (14.3)
Oxaliplatin-based chemotherapy				
	Yes	8	9	17 (60.7)
	No	5	6	11 (39.3)

**Table 2:** Characteristics of individual patients

Case	Age	Sex	Family history of cancer	Disease status	Oxaliplatin-based regimen	Identification of HRR-related gene mutations
1	66	F	Unknown	Metastatic	FOLFIRINOX	No
2	52	F	Gastric cancer (FDR), Colorectal cancer (TDR), Lung cancer (TDR)	Metastatic	FOLFIRINOX	No
3	42	M	Pancreatic cancer (SDR)	Locally advanced	SOX	No
4	64	M	None	Locally advanced	GEMOX	No
5	39	F	Cutaneous cancer (FDR) Esophageal cancer (TRD)	Metastatic	SOX	No
6	64	M	None	Recurrence after adjuvant S-1	SOX	No
7	74	F	Lung cancer (FDR)	Recurrence after adjuvant S-1	SOX	No
8	65	M	Gastric cancer (FDR), Lung cancer (TDR)	Metastatic	SOX	No
9	66	F	Unknown	Recurrence after adjuvant S-1	GEMOX	No
10	55	M	None	Metastatic	FOLFIRINOX	Yes
11	52	M	Brain tumor (FDR) Prostate cancer (TRD)	Locally advanced	FOLFIRINOX	Yes
12	68	M	Gastric cancer (FDR)	Metastatic	FOLFIRINOX	Yes
13	47	M	Pancreatic cancer (FDR)	Metastatic	GEMOX	Yes
14	44	M	None	Recurrence after adjuvant S-1	SOX	Yes
15	65	F	Gastric cancer, Colorectal cancer (FDR)	Metastatic	FOLFOX	Yes
16	81	M	Unknown cancer (FDR)	Metastatic	SOX	Yes
17	57	F	Gastric cancer (FDR)	Recurrence	SOX	Yes
18	65	M	Biliary tract cancer (FDR)	Recurrence	-	No
19	45	M	None	Metastatic	-	No
20	73	M	Colorectal cancer (FDR)	Recurrence after adjuvant S-1	-	No
21	60	F	Pancreatic cancer (FDR)	Recurrence after adjuvant S-1	-	No
22	59	F	Gastric cancer (SDR) Pancreatic cancer (TDR)	Recurrence after adjuvant S-1	-	No
23	67	M	None	Recurrence	-	No
24	60	F	Unknown	Recurrence after adjuvant S-1	-	Yes
25	61	M	Breast cancer (FDR)	Locally advanced	-	Yes
26	67	F	Gastric cancer (FDR)	Locally advanced	-	Yes
27	77	F	Unknown	Locally advanced	-	Yes
28	74	F	None	Locally advanced	-	Yes

FDR, first-degree relative; SDR, second-degree relative; TDR, third-degree relative.

### Completion rate of the NGS-based multiplex gene assay

**Figure 1:**
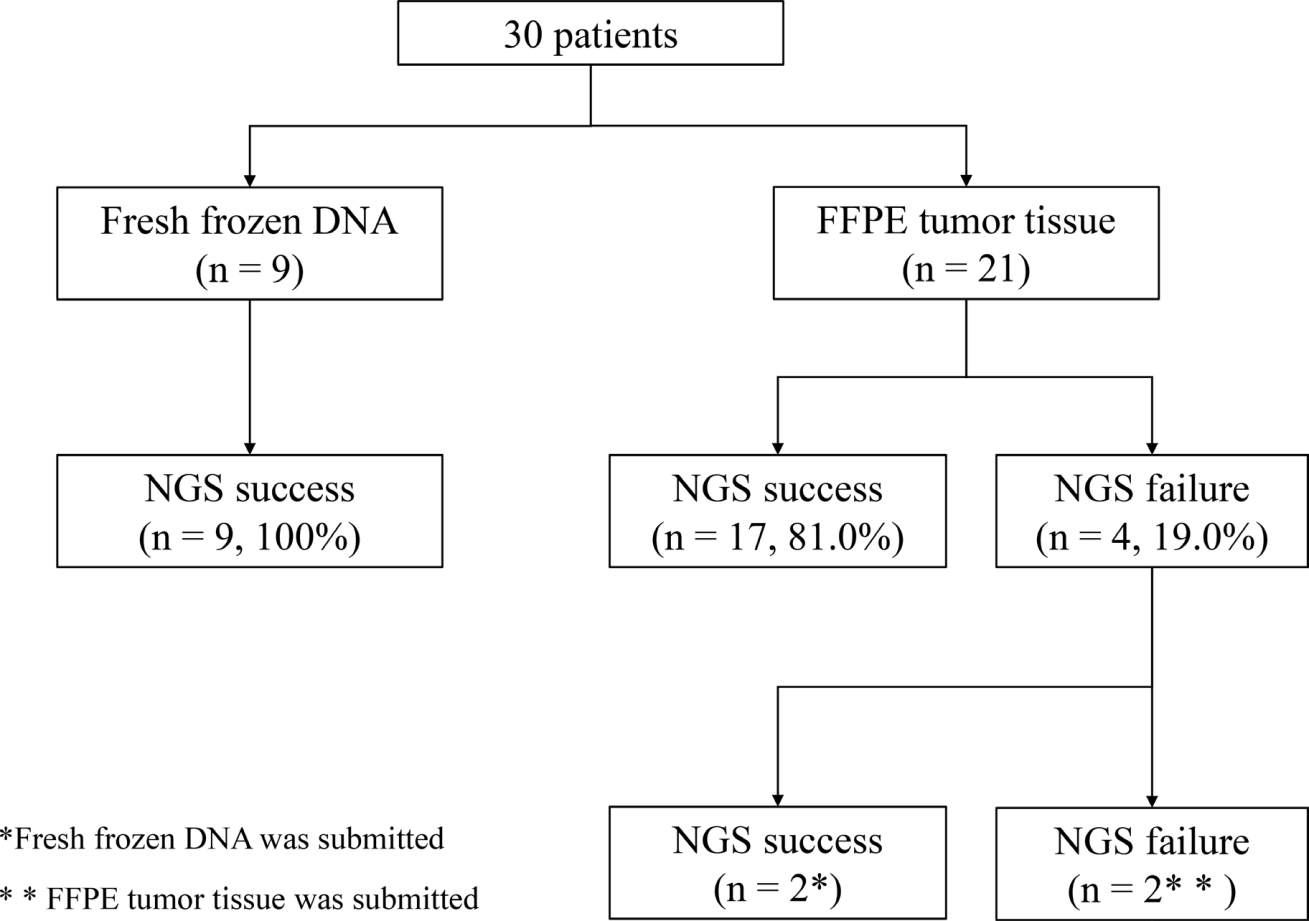
Success rate of multiplex next-generation sequencing (NGS) assay in 30 consecutive patients with pancreatic cancer

The NGS-based multiplex gene assay was performed using archival formalin-fixed paraffin-embedded (FFPE) tumor tissues (*n* = 21) or fresh frozen tumor tissues obtained from liver metastases (*n* = 5), primary sites (*n* = 3), and a lymph node metastasis (*n* = 1). The first NGS assay failed in four patients because of poor DNA quality, and a second assay was successfully completed in two patients using fresh frozen tissue obtained via fine-needle aspiration from a liver metastasis (*n* = 1) and a primary site (*n* = 1). The overall completion rate of the NGS assay was 93.3% (Figure 1).

### Identification of non-synonymous mutations in HRR-related genes

The identified HRR-related gene mutations and their corresponding ID, reported in the *Catalogue of Somatic Mutations in Cancer* (COSMIC) database and the reference single nucleotide polymorphism (rs) ID in dbSNP, are summarized in Table 3. *BRCA2* was the most commonly mutated gene (*n* = 10), followed by *ATM* (*n* = 8), *BRCA1* (*n* = 2), *CHEK2* (*n* = 2), *ATR* (*n* = 1), and *PALB2* (*n* = 1). In total, non-synonymous HRR-related gene mutations were identified in 13 patients (46.4%). Germline allele frequency of each variant in the normal population as reported in the Exome Aggregation Consortium (http://exac.broadinstitute.org/) and Human Genetic Variation Database (http://www.hgvd.genome.med.kyoto-u.ac.jp/) are also summarized in Table 3.

**Table 3:** Identified HRR-related gene mutations

Case	Gene	Mutation	Function	COSMIC ID	dbSNP ID	Germline test	ExAC	ExAc East Asian	HGVD
10	ATR	I774fs	Inactivating mutation	1617015	Not reported	negative	Not reported	Not reported	Not reported
	ATM	L2005V	VUS	Not reported	Not reported	positive	Not reported	Not reported	Not reported
	BRCA2	I1929V	VUS	Not reported	rs79538375	positive	9.6e-04	9.8e-03	Not reported
11	ATM	R2034X	Inactivating mutation	922732	rs532480170	-	1.7e-05	0	Not reported
	ATM	L2426I	VUS	Not reported	Not reported	-	Not reported	Not reported	Not reported
	BRCA1	L52F	VUS	Not reported	rs80357084	-	1.3e-04	1.8e-03	2.5e-03
12	BRCA2	S1989fs	Inactivating mutation	Not reported	rs80359552	-	Not reported	Not reported	Not reported
	BRCA2	N2436I	VUS	Not reported	rs80358955	-	8.2e-06	0	2.1e-03
	CHEK2	R474C	Inactivating mutation	Not reported	rs540635787	-	8.8e-06	1.2e-04	Not reported
13	ATM	R1618X	Inactivating mutation	1350875	Not reported	positive	Not reported	Not reported	Not reported
14	BRCA2	Q3026X	Inactivating mutation	3468418	rs80359159	-	1.7e-05	0	Not reported
15	ATM	L1700fs	Inactivating mutation	Not reported	Not reported	-	Not reported	Not reported	Not reported
16	PALB2	splice site 3350+5G>A	VUS	Not reported	rs587782566	positive	Not reported	Not reported	Not reported
	BRCA2	A2351G	VUS	Not reported	rs80358932	positive	1.3e-04	1.7e-03	1.2e-03
17	BRCA1	R1443X	Inactivating mutation	979730	rs41293455	-	1.6e-05	0	Not reported
24	BRCA2	S871X	Inactivating mutation	Not reported	rs397507634	negative	Not reported	Not reported	Not reported
	BRCA2	splice site 7977-2A>T	Inactivating mutation	Not reported	rs276174899	negative	Not reported	Not reported	Not reported
	BRCA2	V2503I	VUS	Not reported	rs587782191	negative	8.2e-06	Not reported	4.2e-04
	ATM	R2691C	VUS	922745	rs531980488	negative	1.1e-04	7.0e-04	Not reported
25	ATM	V1038X	Inactivating mutation	Not reported	Not reported	-	Not reported	Not reported	Not reported
26	BRCA2	R2318X	Inactivating mutation	Not reported	rs80358920	-	Not reported	Not reported	Not reported
27	BRCA2	I1929V	VUS	Not reported	rs79538375	-	9.6e-04	9.8e-03	0.012
	ATM	V2951I	VUS	Not reported	Not reported	-	Not reported	Not reported	Not reported
28	CHEK2	splice site 1591-1G>A	VUS	Not reported	Not reported	-	Not reported	Not reported	Not reported

VUS, variant of unknown significance; ExAC, Exome Aggregation Consortium; HGVD, Human Genetic Variation Database.

### Family history of cancer

Among the 28 patients evaluated, family history of any cancer and of pancreatic cancer within third-degree relatives was confirmed in 17 (60.7%) and four patients (14.3%), respectively. Of the 13 patients harboring HRR-related gene mutations, only one had a family history of pancreatic cancer (Table 1).

### Efficacy of oxaliplatin-based chemotherapy

**Figure 2:**
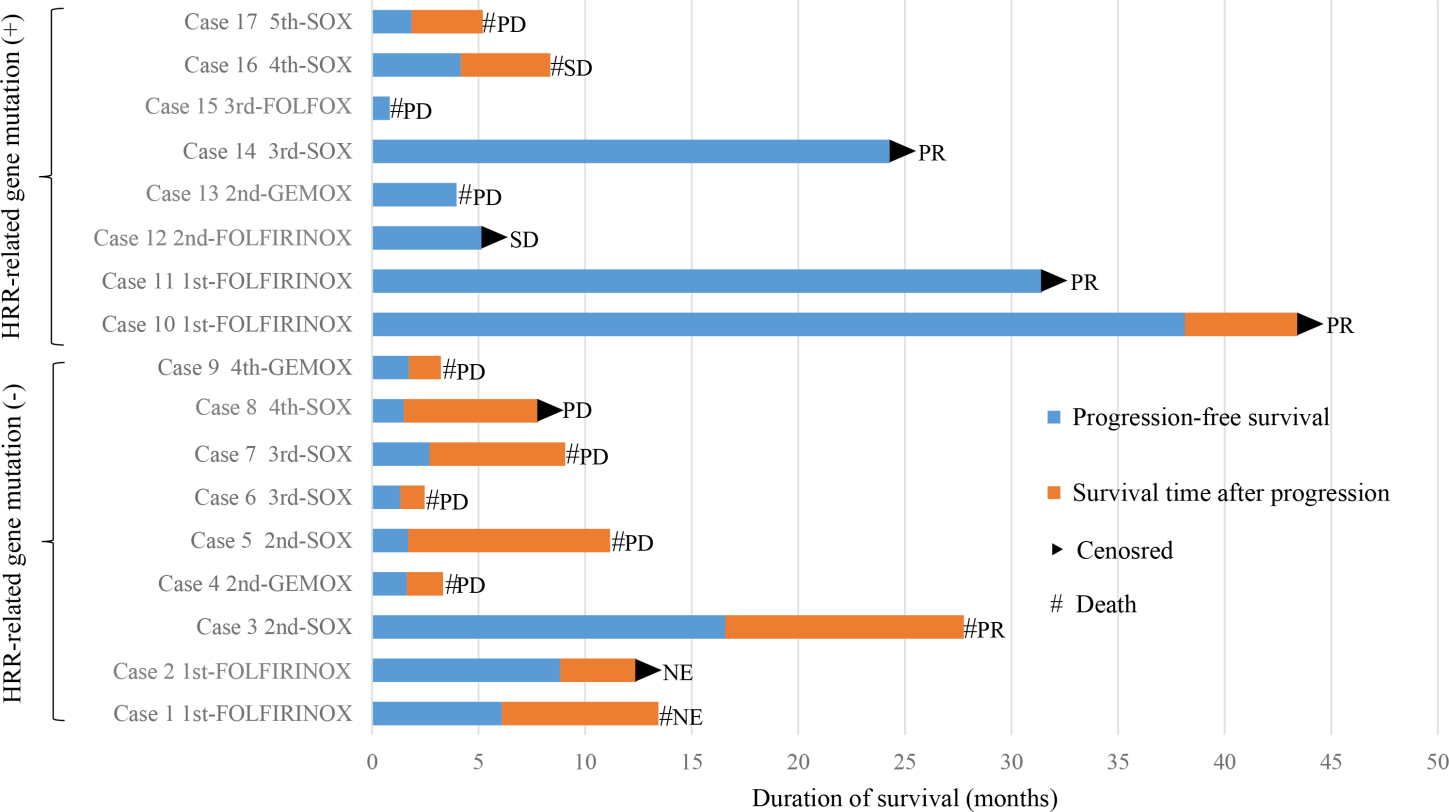
Progression-free survival, survival time after progression, and the response of individual patients who received oxaliplatin-based chemotherapy

**Figure 3:**
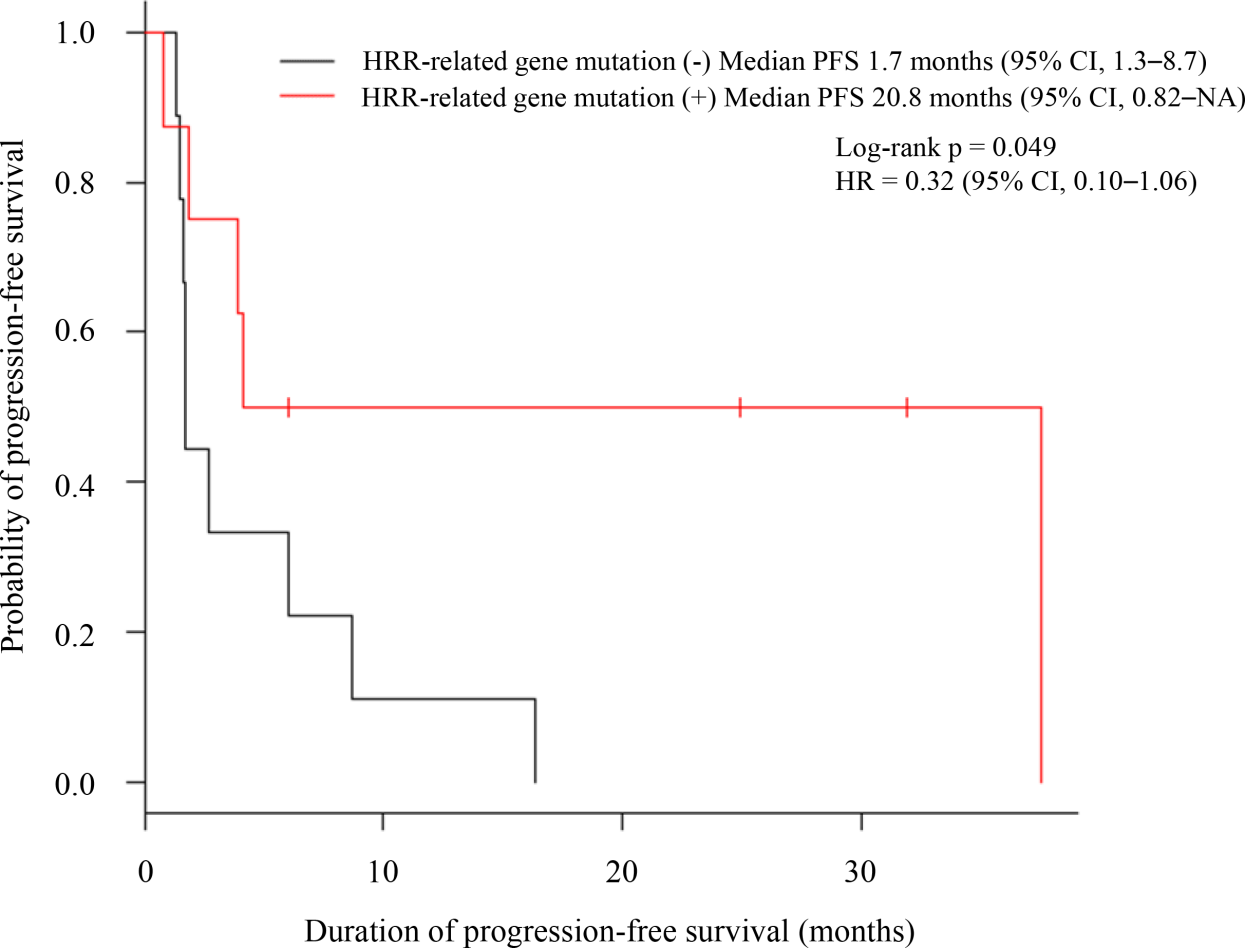
Kaplan–Meier estimates of progression-free survival in patients who received oxaliplatin-based chemotherapy

Of the 28 patients evaluated, 17 received oxaliplatin-based chemotherapy. Treatment regimens are summarized in Table 2. Three radiologic responses and one tumor marker response (CA 19-9 and CEA decrease *>* 60%) were observed in patients harboring HRR-related gene mutations while only one radiologic response was observed in those without such mutations. Progression-free survival (PFS) and survival time after progression in individual patients are shown in Figure 2. Median PFS was significantly longer in patients with HRR-related gene mutations than those without mutations (20.8 months vs. 1.7 months, respectively; *p* = 0.049, Figure 3) and hazard ratio (HR) was 0.32 (95% confidence interval (CI), 0.10–1.06, *p* = 0.061).

## DISCUSSION

In this study, non-synonymous HRR-related gene mutations were identified in 13 (46.4%) of 28 consecutive patients with PDAC who were evaluated using an NGS-based multiplex gene assay covering complete coding exons of 12 HRR-related genes. Among the 13 patients with HRR-related gene mutations, only one (7.6%) had a family history of pancreatic cancer (Table 2). In line with our current results, Shindo et al. recently reported that among 27 patients with pancreatic cancer harboring pathogenic germline mutations in *BRCA1*/*BRCA2*/*ATM*/*PALB2*, only three patients (11.1%) had a family history of pancreatic cancer [14]. These data suggest that prevalence of HRR-related gene mutations is not uncommon, even if a patient has no family history of pancreatic cancer.

Efficacy of oxaliplatin-based chemotherapies such as FOLFIRINOX, FOLFOX, GEMOX, and SOX in pancreatic cancer has been tested in several clinical trials [8, 9, 11, 12]. Among these, only FOLFIRINOX exhibited clear significant benefit over gemcitabine monotherapy (control) in a large randomized clinical trial and has been recommended for a standard treatment regimen [8]. Conversely, SOX failed to exhibit benefit over S-1 monotherapy in a second-line setting in a randomized clinical trial and is not recommended as standard treatment in routine clinical practice [11]. However, in this study, we observed one patient with an inactivating HRR-related gene mutation (*BRCA2* Q3026X) who did not respond to the standard chemotherapy of gemcitabine/nab-paclitaxel but exhibited a partial response to SOX (Figure 2, Case 14). These results suggested that an oxaliplatin-based regimen, which failed to demonstrate a positive result in a clinical trial involving random patients with PDAC, is still beneficial in patients harboring HRR-related gene mutations.

Among 28 patients, 17 received oxaliplatin-based chemotherapy. In total, three of eight patients who harbored HRR-related gene mutations showed a radiologic response to oxaliplatin-based chemotherapy and one showed a CA19-9 and CEA decrease of *>* 60% at 8 weeks, which has been shown to be a predictor of better overall survival [15], whereas the response rate was 11.1% (1/9) among patients without such mutations. Interestingly, two patients with HRR-related gene mutations who received FOLFIRINOX as first-line treatment showed exceptional PFS responses for *>* 24 months (Figure 2, Cases 10 and 11). Because impaired HRR may confer sensitivity to platinum agents and topoisomerase inhibitors [5, 16, 17], patients with HRR-related gene mutations may experience greater benefit from FOLFIRINOX treatment.

On the other hand, four patients with HRR-related gene mutations showed neither radiologic nor tumor marker response to oxaliplatin-based chemotherapy. One patient (Case 13) refused to continue chemotherapy after the first cycle of GEMOX due to its toxicity. The other three patients (Case 15, 16, 17), received oxaliplatin-based chemotherapy as third or later line treatment and this might have influenced the poor response. For example, Case 15 was obliged to discontinue chemotherapy after the first cycle of FOLFOX due to rapid deterioration of uncontrollable ascites. Therefore, to derive maximum benefit from oxaliplatin-based chemotherapy in patients with HRR-related gene mutations, it appears to be relevant to attempt this regimen at an earlier treatment time-point under better patient general status.

Pathogenic germline *BRCA1/2* mutations are listed among the genes recommended for reporting of secondary findings by the American College of Medical Genetics and Genomics (ACMG) [18], and we follow this recommendation in daily clinical practice. In this study, suspected pathogenic germline *BRCA1/2* mutations that met the ACMG recommendations were found in five patients (Table 3, Cases 12, 14, 17, 24, and 26). However, except for case 24, four patients did not undergo the germline test primarily because the test results would not affect their own cancer treatment. We were able to conduct the additional germline DNA test in four patients who provided informed consent, and five of 10 HRR-related mutations were confirmed to be germline mutations (Table 3).

Limitations of this study included a limited sample size, and differences in lines of treatment and oxaliplatin-based chemotherapy regimens among the patients. In addition, we could not eliminate the possibility that mutations identified in nine patients who did not undergo the germline test may have been derived from germline DNA.

In summary, the status of HRR-related gene mutations was positively associated with oxaliplatin-based chemotherapy in patients with PDAC. Monitoring HRR-related genes using an NGS assay might be useful in selecting PDAC patients potentially sensitive to oxaliplatin-based chemotherapy. We are currently planning a prospective trial to verify the results reported here and further explore precision medicine in the field of pancreatic cancer.

## MATERIALS AND METHODS

### Patients

A total of 30 consecutive patients with histologically confirmed PDAC who underwent an NGS-based multiplex assay (OncoPrime^TM^) at Kyoto University Hospital between April 2015 and July 2017 were eligible for this study.

### NGS-based multiplex assay (OncoPrime^TM^)

An NGS-based multiplex assay (OncoPrime^TM^) covers complete coding exons of 215 cancer-related genes and rearrangements in 17 frequently rearranged genes (Supplementary Table 1) [13, 19]. DNA extracted from archived FFPE tissue samples or fresh frozen tissue samples was used for this assay. NGS was performed in a CLIA-certified laboratory using Illumina HiSeq 2500 by EA Genomics (Morrisville, North Carolina, United States).

### Identification of HRR-related gene mutations

Twelve HRR-related genes included in OncoPrime^TM^ (*ATM*, *ATR*, *BAP1*, *BRCA1*, *BRCA2*, *BLM*, *CHEK1*, *CHEK2*, *FANCA*, *MRE11A*, *PALB2*, and *RAD51*) were evaluated in this study. Variant calling was performed using variant calling software (VarPROWL) in a CLIA-certified laboratory by EA Genomics as previously reported [13] based on the following workflow:

Step 1: Remove all silent mutations in non-reference alleles, retaining mutations that are missense, are nonsense, or involve splicing junctions.

Step 2: Remove all non-reference alleles that appear in *>* 1% of the population (high minor allele frequency) because they are likely germline events.

Step 3: Remove all non-reference alleles with allele frequencies of *<* 4% and *>* 95%. This was performed because the limit of detection was 4% and alleles with > 95% frequency were most likely germline DNA because the sample material had at least 20% tumor content.

Step 4: The identified mutations were prioritized based on their presence in the following databases: Online Mendelian Inheritance in Man (https://www.omim.org/), ClinVar (https://www.ncbi.nlm.nih.gov/clinvar/), Clinical Trials.gov (https://clinicaltrials.gov/), Drug Bank (https://www.drugbank.ca/), COSMIC (http://cancer.sanger.ac.uk/cosmic), and the Cancer Genome Atlas (https://cancergenome.nih.gov/).

After the filtering process mentioned above, non-synonymous mutations including variants of unknown significance were considered HRR-related gene mutations.

### Efficacy of oxaliplatin-based chemotherapy

Oxaliplatin-based chemotherapy includes oxaliplatin, irinotecan, fluorouracil, and *l*-leucovorin (FOLFIRINOX); gemcitabine and oxaliplatin (GEMOX); and S-1 and oxaliplatin (SOX) [8, 11, 12]. Standard doses and schedules of the regimens were adjusted at the discretion of the treating physicians based on incidence of adverse events and individual patient general status. Clinical data were retrieved using a prospective cohort database system (CyberOncology^®^; Cyber Laboratory Inc., Tokyo, Japan) and electronic medical records.

### Statistical analysis

Objective response was assessed based on the Response Evaluation Criteria in Solid Tumors (RECIST) version 1.1 [20]. PFS was defined as the interval between date of initiation of oxaliplatin-based chemotherapy and date of disease progression or death due to any cause. Survival time after disease progression was defined as the interval between date of disease progression and death. Patients not experiencing disease progression or death were censored at the last follow-up visit. Median PFS was estimated using the Kaplan-Meier method, and differences were compared using the log-rank test. The hazard ratio and 95% confidence interval were calculated using Cox regression models. The data cutoff date was November 30, 2017. Statistical analyses were performed using R version 3.4.1.

### Ethics

This study was approved by the Ethics Committee of Kyoto University Graduate School of Medicine (G692) and conducted in accordance with the Declaration of Helsinki. All patients provided written informed consent for the use of genomic and clinical data for research purposes.

## SUPPLEMENTARY MATERIALS TABLE



## Abbreviations

CLIA: Clinical Laboratory Improvement Amendment
COSMIC: catalogue of somatic mutations in cancer
GEMOX: gemcitabine and oxaliplatin
FOLFIRINOX: oxaliplatin, irinotecan, fluorouracil, and leucovorin
FOLFOX: oxaliplatin, fluorouracil, and leucovorin
HRR: homologous recombination repair
NGS: next-generation sequencing
OS: overall survival
PD: progressive disease
PDAC: pancreatic ductal adenocarcinoma
PFS: progression-free survival
PR: partial response
RECIST: Response Evaluation Criteria in Solid Tumors
SD: stable disease
SNP: single nucleotide polymorphism
SOX: S-1 and oxaliplatin
