# Supplementary material for: Association between homologous recombination repair gene mutations and response to oxaliplatin in pancreatic cancer
Source: Oncotarget. 2018 Apr 13;9(28):19817–25. doi: 10.18632/oncotarget.24865 (PMC5929428; doi:10.18632/oncotarget.24865)
Supplement: Supplementary file 1 [file oncotarget-09-19817-s001.pdf]

Association between homologous recombination repair gene mutations and response to oxaliplatin in pancreatic cancer

SUPPLEMENTARY MATERIALS

Supplementary Table 1: Genes sequenced in OncoPrime™

ABL	CARD11	DNMT3A	GATA2	MAP2K2	NOTCH3	PTEN	STAT3
ABL2	CASP8	DPYD	GATA3	MAP2K4	NOTCH4	PTPN11	STK11
ACVR1B	CBL	EGFR	GLI1	MAP3K1	NPM1	RAD50	SUFU
AKT1	CCND1	EP300	GNA11	MAPK1	NRAS	RAD51	TERT
AKT2	CCND2	ERBB2	GNAQ	MDM2	NTRK1	RAF1	TET2
AKT3	CCND3	ERBB3	GNAS	MDM4	NTRK2	RB1	TGFBR2
ALK	CCNE1	ERBB4	GRIN2A	MED12	NTRK3	RET	TNFAIP3
APC	CDC73	ERCC1	H3F3A	MEN1	PALB2	RICTOR	TOP1
AR	CDH1	ERCC2	HNF1A	MET	PARP1	RNF43	TOP2A
ARAF	CDK4	ERCC3	HRAS	MITF	PAX5	ROS1	TP53
ARID1A	CDK6	ERG	IDH1	MLH1	PBRM1	RPTOR	TP63
ARID1B	CDKN2A	ERRFI1	IDH2	MLL	PDGFRA	RSPO2	TP73
ASXL1	CDKN2B	ESR1	IGF1R	MPL	PDGFRB	RSPO3	TPMT
ATM	CEBPA	EZH2	IGF2R	MRE11A	PDK1	RUNX1	TRAF7
ATR	CHEK1	FAM123B	IKZF1	MSH2	PGR	SETD2	TSC1
ATRX	CHEK2	FANCA	IL7R	MSH6	PHF6	SF3B1	TSC2
AURKA	CREBBP	FBXW7	INSR	MTHFR	PIK3CA	SMAD2	TSHR
AURKB	CRLF2	FGFR1	JAK1	MTOR	PIK3CG	SMAD3	TYMS
AXIN1	CSF1R	FGFR2	JAK2	MYC	PIK3R1	SMAD4	U2AF1
BAP1	CTNNA1	FGFR3	JAK3	MYCN	PIK3R2	SMARCA4	UGT1A1
BCL2	CTNNB1	FGFR4	KDM6A	MYD88	PIK3R5	SMARCB1	VHL
BCOR	CYP1A2	FLT1	KDR	NBN	PMS1	SMO	VKORC1
BLM	CYP2C19	FLT3	KIT	NF1	PMS2	SOCS1	WRN
BRAF	CYP2C9	FLT4	KLF4	NF2	PPP2R1A	SRC	WT1
BRCA1	CYP2D6	FOXL2	KRAS	NFE2L2	PRDM1	SRSF2	XPC
BRCA2	DAXX	G6PD	MAML1	NOTCH1	PTCH1	STAG2	XRCC1
BTK	DDR2	GATA1	MAP2K1	NOTCH2	PTCH2	STAT1	
**Genes targeted for rearrangement detection**							
ALK	EGFR	ETV4	ETV6	MLL	PDGFRB	RARA	ROS1
BCR	ETV1	ETV5	EWSR1	PDGFRA	RAF1	RET	TMPRSS2
BRAF							
